# Importation of Mumps Virus Genotype K to China from Vietnam

**DOI:** 10.3201/eid2404.170591

**Published:** 2018-04

**Authors:** Wei Liu, Lili Deng, Xianyu Lin, Ximing Wang, Yuyan Ma, Qiuyun Deng, Xiaohua Xue, Ge Zhong, Li Jin

**Affiliations:** Guangxi Zhuang Autonomous Region Center for Disease Prevention and Control, Nanning, China (W. Liu, L. Deng, Y. Ma, Q. Deng, G. Zhong);; Shuikou Entry–Exit Inspection and Quarantine Bureau, Longzhou, China (X. Lin, X. Xue);; Longzhou County Center for Disease Prevention and Control, Longzhou (X. Wang); Public Health England, London, UK (L. Jin)

**Keywords:** mumps, mumps virus, viruses, genotype K, importation, parotid glands, Vietnam, China

## Abstract

During May–August 2016, mumps virus genotype K was detected in 12 Vietnam citizens who entered China at the Shuikou border crossing and 1 girl from China. We provide evidence that mumps genotype K is circulating in Vietnam and was imported to China from Vietnam.

Mumps is an acute viral illness characterized by swelling of the parotid glands. The disease is highly contagious and shows nonspecific symptoms (e.g., headache and fever). However, in some cases complications, such as aseptic meningitis, orchitis, encephalitis, and deafness, might occur (*1*).

Mumps can be prevented by appropriate vaccination. Mumps virus–containing vaccine (e.g., MMR vaccines for measles, mumps, and rubella) has been given to children 18–24 months of age in China since 2008. However, this vaccine has not been included in immunization schedules in Vietnam (*2*). The Shuikou border crossing is located between TàLùng in CaoBằng Province, Vietnam, and Shuikou in Guangxi Province, China (Technical Appendix Figure 1). Approximately ≈1,500 persons/day cross the border between Vietnam and China to conduct business or receive healthcare. More than 90% of these persons are from Vietnam, many of whom visit clinics in Shuikou and return home the same day. We report importation of mumps virus genotype K to China from Vietnam.

## The Study

On May 15, 2016, a 7-year-old boy from Vietnam with bilateral parotid gland swelling was observed at the border crossing and considered to have a suspected case of mumps. Over the next 4 months, 11 other patients from TàLùng (Technical Appendix Figure 1) with suspected mumps were reported to the Longzhou County Center of Disease Prevention and Control (CDC) in China. None of these 12 patients had traveled outside CaoBằng Province, Vietnam, during the month before onset of infection. However, 7 patients reported contact with persons who had mumps-like illness.

On July 15, 2016, an 11-year-old girl from China with fever and unilateral parotid gland swelling was clinically confirmed as having mumps (Table). She was living in a village near the Shuikou border crossing, and her father and other villagers used this border crossing almost daily to conduct business, which suggested that she might be epidemiologically linked to the Vietnam patients. None of the 13 patients had received a mumps vaccine. We obtained data for all 13 patients (Table).

**Table:** Characteristics of 13 mumps patients during importation of mumps virus genotype K to China from Vietnam, 2016*

Patient no.	Age, y/sex	Date of illness onset	Date of sample collection	Parotid gland swelling	Body temperature, °C	Virus isolation result	Mumps virus strain
1	7/M	May 15	May 15	Bilateral	37.3	+	MuVi/CaoBang.VNM/20.16/1[K]
2	9/F	May 16	May 21	Bilateral	36.8	–	MuVs/CaoBang.VNM/21.16/1[K]
3	4/M	May 16	May 22	Bilateral	37.5	–	MuVs/CaoBang.VNM/21.16/2[K]
4	5/M	Jun 4	Jun 4	Unilateral	37.5	+	MuVi/CaoBang.VNM/23.16/1[K]
5	4/M	Jun 6	Jun 6	Bilateral	37.0	+	MuVi/CaoBang.VNM/24.16/1[K]
6	5/M	Jun 13	Jun 14	Unilateral	37.5	+	MuVi/CaoBang.VNM/25.16/1[K]
7	6/M	Jun 13	Jun 14	Unilateral	38.0	+	MuVi/CaoBang.VNM/25.16/2[K]
8	5/F	Jun 17	Jun 19	Bilateral	37.0	+	MuVi/CaoBang.VNM/25.16/3[K]
9†	11/F	Jul 15	Jul 20	Unilateral	38.8	–	MuVs/Guangxi.CHN/29.16/1[K]
10	10/M	Jul 15	Jul 15	Unilateral	38.6	–	MuVs/CaoBang.VNM/29.16/1[K]
11	27/F	Aug 1	Aug 3	Unilateral	37.0	+	MuVi/CaoBang.VNM/32.16/1[K]
12	23/F	Aug 1	Aug 3	Bilateral	36.8	+	MuVi/CaoBang.VNM/32.16/2[K]
13	30/M	Aug 4	Aug 8	Bilateral	36.5	–	MuVs/CaoBang.VNM/32.16/3[K]

*All patients had positive results for mumps virus by PCR. +, positive; –, negative. †Patient in China.

Throat swab specimens were collected from patients and immediately transported on icepacks to the Guangxi CDC. RNA was extracted from original samples and supernatants of Vero/hSLAM-cell cultures (*3*) by using the Viral RNA Mini Kit (QIAGEN, Valencia, CA, USA). We amplified extracted RNA by using the SuperScript-III Platinum One-Step Reverse Transcription PCR System (Invitrogen, Waltham, MA, USA) and primers for the small hydrophobic (SH) gene and hemagglutinin–neuraminidase (HN) gene as described (*4*,*5*). All throat swab specimens were positive for mumps virus by reverse transcription PCR. Mumps virus isolation was achieved for 8 (61.5%) of 13 specimens.

**Figure 1:**
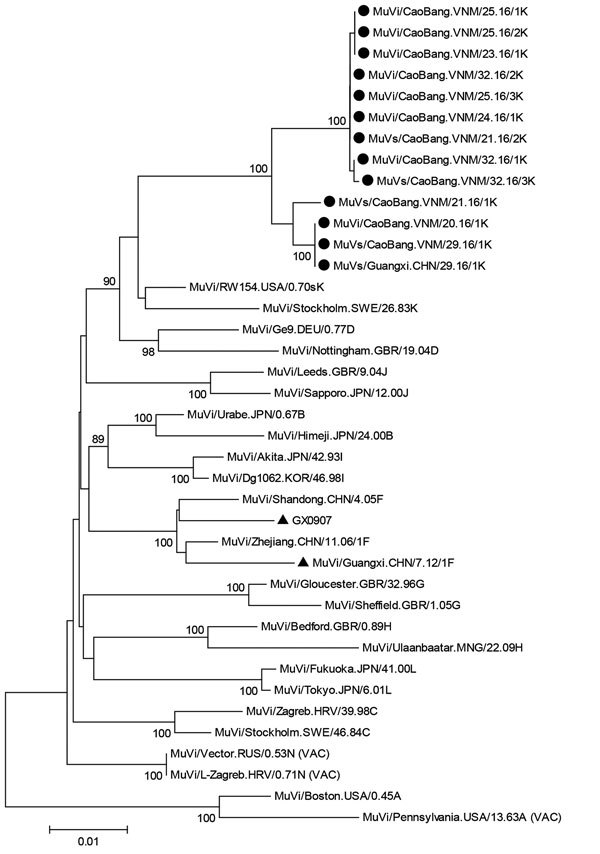
Phylogenetic tree of hemagglutinin–neuraminidase genes of 13 isolates of mumps virus genotype K from 1 patient from China and from 12 patients from Vietnam (solid black circles) compared with reference isolates. Solid black triangles indicate F strains isolated in Guangxi Province, China. The tree was constructed by using the neighbor-joining method in MEGA6 software (*7*). The Kimura-2 parameter model was used, and robustness of internal branches was determined by using 500 bootstrap replications. Numbers along branches are bootstrap values. Scale bar indicates nucleotide substitutions per site. MuV, mumps virus.

PCR products were sequenced by Life Technologies (Shanghai, China). We performed analyses of nucleotide sequences of SH and HN genes and deduced amino acid sequences by using BioEdit (*6*) and the neighbor-joining method in MEGA6 software (*7*). In accordance with World Health Organization mumps virus nomenclature (*8*), viruses were named and assigned a genotype on the basis of comparison with 12 reference strains and a high bootstrap score. Sequences formed 2 distinct clusters; both clusters had greatest homology with genotype K reference strains Stockholm.SWE/26.83/1[K] and RW154.USA/0.70s[K]. The SH gene homologies were 93.0%–94.3% with Stockholm.SWE/26.83/1[K] and 93.9%–95.8% with RW154.USA/0.70s[K]. Greatest heterogeneity was 15.6% with genotype A (Technical Appendix Figure 2). A similar pattern was observed by additional phylogenetic analysis with the HN gene (Figure 1), recommended by the World Health Organization (*8*). These analyses confirmed that all viruses had genotype K.

**Figure 2:**
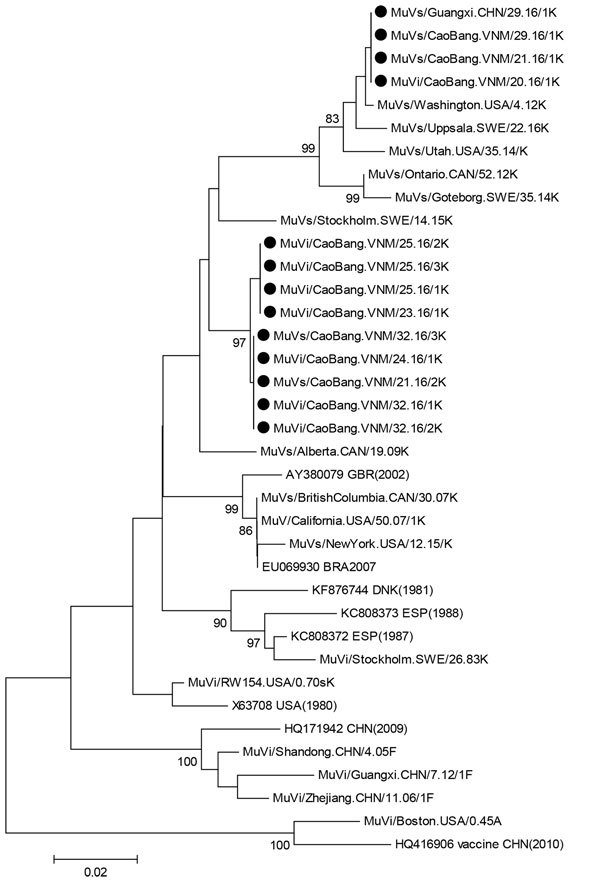
Phylogenetic tree of small hydrophobic genes of 13 isolates of mumps virus genotype K isolated in China from 1 patient from China and 12 patients from Vietnam (solid black circles) compared with reference isolates. The tree was constructed by using the neighbor-joining method in MEGA6 software (*7*). The Kimura-2 parameter model was used, and robustness of internal branches was determined by using 500 bootstrap replications. Numbers along branches are bootstrap values. Scale bar indicates nucleotide substitutions per site. MuV, mumps virus.

We performed further analysis of the SH gene to evaluate diversity of genotype K by construction of a phylogenetic tree that included 17 genotype K sequences from GenBank; 2 F strains detected in Guangxi Province during 2009–2012; reference strains of genotypes F, K, and A; and the mumps vaccine (S79/Jeryl Lynn, HQ416906) used in China. Homologies between the 2 clusters and other K strains ranged from 94.9% to 100% (Figure 2). Cluster 1, which contains the sequence obtained from the patient in China, is only 1 nt different from the K strain detected in the United States in 2012 (Washington.USA/4.12). This cluster is also closer to K strains detected more recently (2012–2016). Cluster 2 contains the remaining sequences from Vietnam, and this cluster is closely related to the K strain reported in 2009 in Canada (Alberta.CAN/19.09[K]). The genotype F virus identified in Guangxi (Guangxi. CHN/7.12[F]) showed a 9.5%–11.1% difference from the 2 K clusters. All sequences were submitted to GenBank (accession nos. KX622738–KX622745, KX671152, KX965999–KX966002, and KX966004–KX966016).

We compared amino acid sequences of the SH gene with sequences of other genotype K strains and found 5-aa differences between cluster 1 and cluster 2 isolates. The amino acid sequence of the Washington.USA/4.12 strain was identical to those in cluster 1, whereas strains in cluster 2 were unique for all amino acid sequences analyzed (Technical Appendix Figure 3).

## Conclusions

Laboratory investigations suggested an ongoing mumps outbreak in Vietnam. Further supportive information was obtained from the Official News Website of the government of Vietnam (*9*–*12*). During January–May 2016, >600 mumps cases were reported, which was 10 times higher than during the same period in 2015. The mumps outbreak occurred initially in southern Vietnam and quickly spread to northern provinces. In June, 1 hospital in the middle northern region had 50 patients with mumps, of whom 5 had encephalitis as a complication (*12*). The mumps epidemic became more severe in April in the northern region, which includes CaoBằng Province, where the Shuikou border crossing is located. TàLùng, Vietnam, and Shuikou, China, in this region are separated by the river Sông-băc-vọng/Shuikou (Vietnamese/Chinese name) River. Mumps vaccination is not part of the Vietnam national immunization program (*2*), and a shortage of the mumps vaccine has been confirmed by the government of Vietnam.

Because of lack of laboratory data for Vietnam, detection of mumps outbreaks by routine laboratory surveillance has not been established in this country. Patients identified in this report were probably linked to outbreaks described on the basis of timing and residence. We confirm that mumps virus genotype K is circulating in Vietnam, which is consistent with a report that genotype K is still circulating (*13*), and is supported by evidence that 2 recent genotype K strains, 1 from Canada (Ontario.CAN/52.12[K]) and 1 from the United States (Washington.USA/4.12[K]), were imported from Vietnam (R.J. McNall et al., pers. comm.).

The latest summary of global distribution of mumps genotypes (*8*,*13*) showed that genotype F has been circulating in China since 1995. Until now, genotype K was not identified in China. Mumps virus genotype K detected in the patient from China was identical to 1 of the Vietnam genotype K variants, suggesting that transmission of this genotype into China was caused by an importation from Vietnam. No further transmission has been identified in China, suggesting that vaccination coverage is high in the hometown of the child, although some cases might have been missed because up to 30% of mumps infections can be asymptomatic.

The lack of a strong global surveillance system makes understanding transmission patterns of mumps challenging. We provide evidence that mumps genotype K is circulating in Vietnam, and the sequence information presented confirms epidemiologic links between cases. These data increase understanding of the distribution of mumps virus genotypes and highlight the need for enhanced surveillance of infectious diseases at all border crossings between Vietnam and China.

Technical Appendix Additional information on importation of mumps virus genotype K to China from Vietnam.
