# Supplementary material for: Importation of Mumps Virus Genotype K to China from Vietnam
Source: Emerg Infect Dis. 2018 Apr;24(4):774–8. doi: 10.3201/eid2404.170591 (PMC5875267; doi:10.3201/eid2404.170591)
Supplement: Technical Appendix — Additional information on importation of mumps virus genotype K to China from Vietnam. [file 17-0591-Techapp-s1.pdf]

Article DOI: https://doi.org/10.3201/eid2404.170591

Importation of Mumps Virus Genotype K to China from Vietnam

Technical Appendix

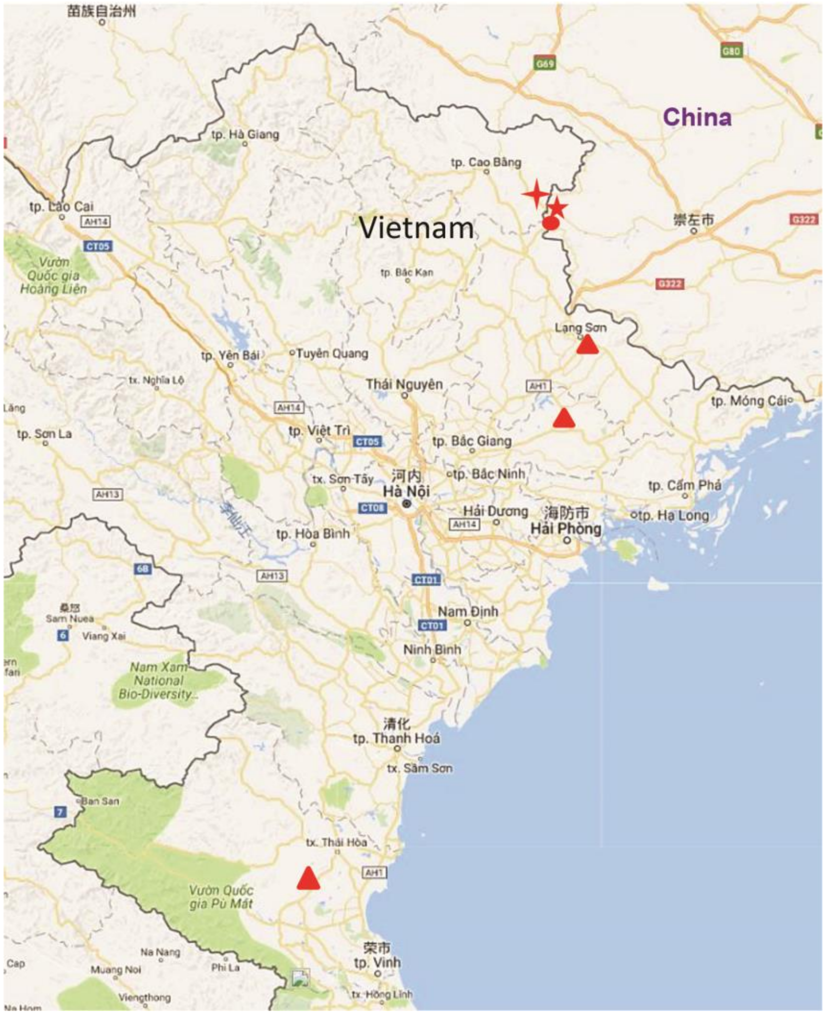

Technical Appendix Figure 1. Locations of recent mumps outbreaks in Vietnam and the border crossing between China and Vietnam. Map was downloaded from Google Maps (https://www.google.com/maps). Red star indicates the Shiukou border crossing between Vietnam and China, where the 12 patients from Vietnam with suspected mumps were reported. Red cross indicates Tà Lùng where the Vietnamese patients resided. The red circle indicates Genyi where the Chinese patient was identified. Red triangles indicate locations of recent mumps outbreaks (Tỉnh Hải Dương, Tỉnh Lạng Sơn, and Tỉnh Nghệ An) as reported by incountry news agencies (https://www.google.com/maps/place/Vietnam/@20.7843144,104.6316833,8.04z/data = !4m5!3m4!1s0x31157a4d736a1e5f:0xb03bb0c9e2fe62be!8m2!3d14.058324!4d108.277199).

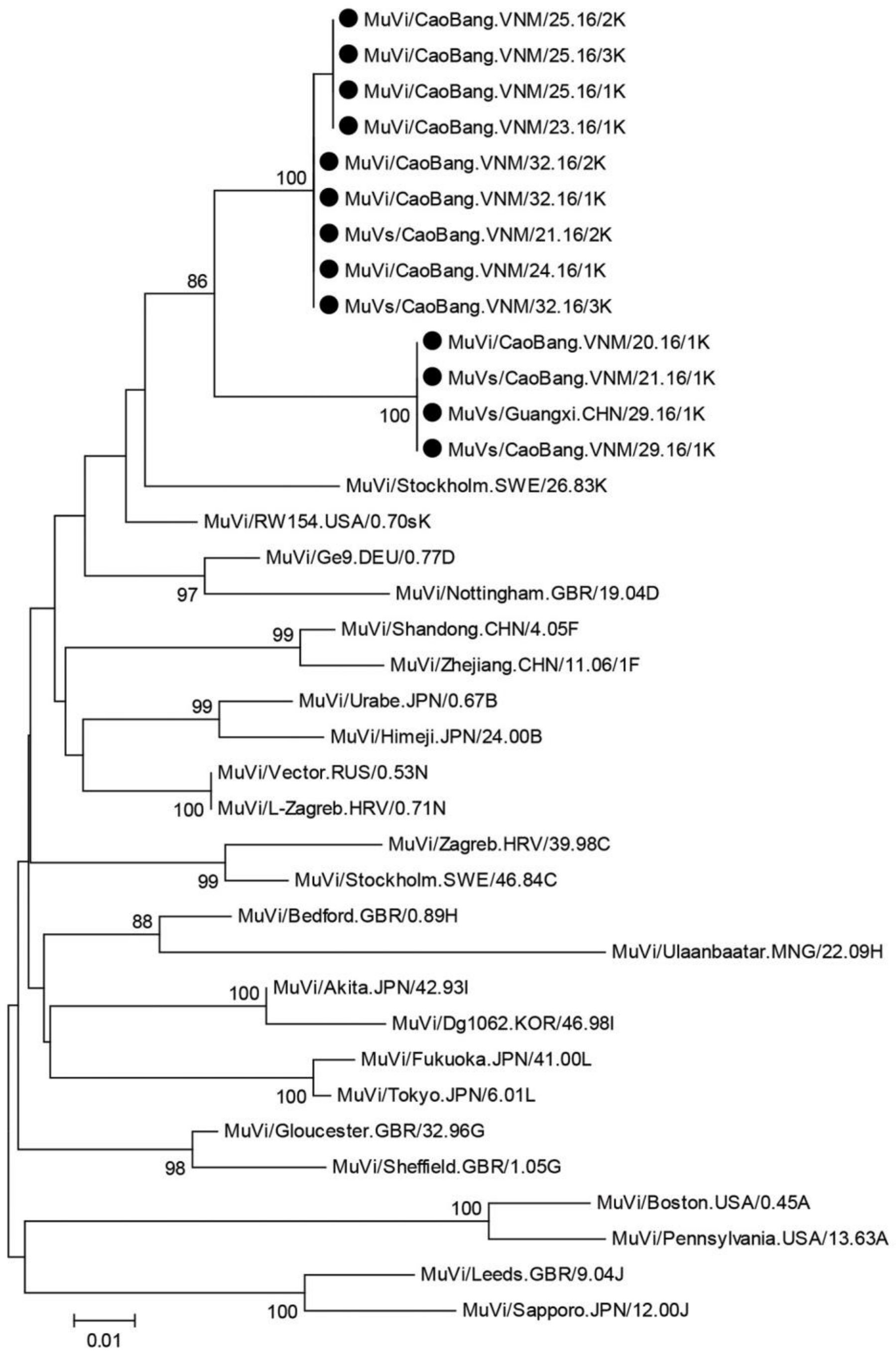

Technical Appendix Figure 2. Phylogenetic tree of small hydrophobic genes of 13 mumps virus strains isolated in China and Vietnam compared with reference isolates. Solid black circles indicate MuV K strains isolated in this study. The tree was constructed by using the neighbor-joining method in MEGA6 software (*1*).The Kimura-2 parameter model was used, and robustness of internal branches was determined by using 500 bootstrap replications. Numbers along branches are bootstrap values. Scale bar indicates nucleotide substitutions per site. MuV, mumps virus.

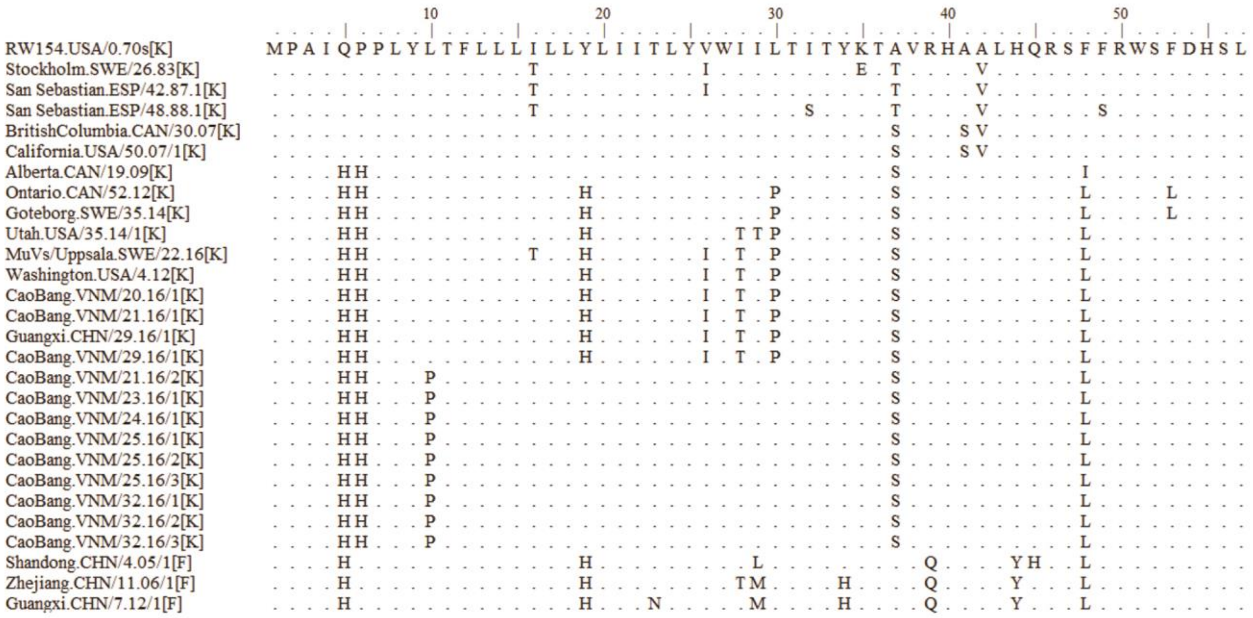

Technical Appendix Figure 3. Sequence alignment based on the predicted amino acids of small hydrophobic genes of mumps virus genotype K strains. A total of 21 genotype K strains, including 12 isolated in this study, and 3 genotype F strains are listed for comparison. Dots indicate sequence identity.

Reference

1. Tamura K, Stecher G, Peterson D, Filipski A, Kumar S. MEGA6: Molecular Evolutionary Genetics Analysis version 6.0. Mol Biol Evol. 2013;30:2725–9. PubMed http://dx.doi.org/10.1093/molbev/mst197
